# Increased Inflammatory Response in Cytomegalovirus Seropositive Patients with Alzheimer’s Disease

**DOI:** 10.1371/journal.pone.0096779

**Published:** 2014-05-07

**Authors:** Gabriel Westman, David Berglund, Johan Widén, Martin Ingelsson, Olle Korsgren, Lars Lannfelt, Dag Sehlin, Anna-Karin Lidehall, Britt-Marie Eriksson

**Affiliations:** 1 Department of Medical Sciences, Uppsala University, Uppsala, Sweden; 2 Department of Immunology, Genetics and Pathology, Uppsala University, Uppsala, Sweden; 3 Department of Public Health and Caring Sciences, Uppsala University, Uppsala, Sweden; University of Leipzig, Germany

## Abstract

Alzheimer’s disease (AD) has been associated with increased local inflammation in the affected brain regions, and in some studies also with elevated levels of proinflammatory cytokines in peripheral blood. Cytomegalovirus (CMV) is known to promote a more effector-oriented phenotype in the T-cell compartment, increasing with age. The aim of this study was to investigate the inflammatory response of peripheral blood mononuclear cells (PBMCs) from AD patients and non-demented (ND) controls. Using a multiplex Luminex xMAP assay targeting GM-CSF, IFN-γ, IL-1β, IL-2, IL-4, IL-5, IL-6, IL-8, IP-10 and TNF-α, cytokine profiles from PBMCs were analysed after stimulation with anti-CD3/CD28 beads, CMV pp65 peptide mix or amyloid β (Aβ) protofibrils, respectively. CMV seropositive AD subjects presented with higher IFN-γ levels after anti-CD3/CD28 and CMV pp65 but not after Aβ stimulation, compared to CMV seropositive ND controls. When analysing IFN-γ response to anti-CD3/CD28 stimulation on a subgroup level, CMV seropositive AD subjects presented with higher levels compared to both CMV seronegative AD and CMV seropositive ND subjects. Taken together, our data from patients with clinically manifest AD suggest a possible role of CMV as an inflammatory promoter in AD immunology. Further studies of AD patients at earlier stages of disease, could provide better insight into the pathophysiology.

## Introduction

Alzheimer’s disease (AD) is, besides the well-described neuropathologic changes with extracellular plaques of amyloid-β (Aβ) and intracellular neurofibrillary tangles (NFT) of tau, characterised by a state of local inflammation in the affected brain regions [1], [2]. Whether patients with AD also have increased systemic inflammation is more controversial. Elevated peripheral levels of proinflammatory cytokines in AD subjects have been found in some studies but not in others [3]. In addition, offspring with a parental history of AD present with a more proinflammatory cytokine profile than those without [4] and patients with AD seem to deteriorate in cognitive capacity when challenged with inflammatory events [5].

The most common form of AD, sporadic late-onset disease, has multiple genetic, vascular and psychosocial risk factors with the *ApoE ε4* genotype being the strongest known genetic predictor [6], [7]. However, it is not completely clear how these factors interact with Aβ in the pathogenesis. Several studies have been addressed to investigate the role of Aβ both in the non-pathological situation and in the pathological processes [8]. There is now increasing evidence that large soluble pre-fibrillar forms of Aβ, so-called protofibrils, are more neurotoxic than monomers [9], [10].

The general role of inflammation in the process of aging has been extensively investigated and developed into a concept of *inflammaging*, suggesting causality between systemic inflammation and the onset and progression of several age-related diseases [11]. Recently, a connection between systemic inflammatory biomarkers, brain microstructure and visuospatial ability has also been found [12].

As many infectious agents are known to cause inflammation through the human immune response, it has been suggested that an accumulation of chronically persistent infections throughout life may contribute to the increase in systemic inflammatory biomarkers that is seen in the elderly [13], [14]. A correlation between infection, systemic inflammation and risk of age-related diseases has been shown for some chronic infections, such as HIV [15], but it remains unclear whether this is also applicable to more common infections, such as human herpes viruses [16]. Several previous studies have shown an association between AD, *ApoE ε4* and herpes simplex virus type 1 (HSV-1) infection or reactivation [17]–[21]. Moreover, evidence suggest that there might be a cumulative cognitive effect of infection with multiple agents in the herpes virus family, including cytomegalovirus (CMV) [22]. Studies addressing the presence of herpes viruses in brain tissue show that HSV-1, HHV-6 and EBV are more often detected in AD, whereas CMV is more frequently found in vascular dementia [23]–[25]. Recently, a study has reported a connection between CMV IgG levels, density of NFT and amyloid load in AD patients and also a correlation between CMV seropositivity, IFN-γ in cerebrospinal fluid (CSF) and NFT density [26]. However, as infection with herpes viruses are related to socio-demographic and behavioural factors [27], there could be confounding factors causing epidemiologic correlations between infection and cognitive performance without direct causality.

CMV is a betaherpesvirus causing a chronically persistent infection throughout life. It is known to promote profound changes in the T-cell compartment with CD8 clone expansions and effector-oriented differentiation [28]–[30]. HLA-A02 positive subjects generally present with a strong and reliable cellular immune response against epitopes in the CMV internal matrix protein pp65 [31]. Whether CMV infection also affects the cellular immune response against Aβ, possibly connecting to the AD pathogenesis, is not yet known as previous studies has been inconclusive [32]. We have previously shown that AD patients present with a significantly lower proportion of CMV-specific CD8 T-cells than non-demented (ND) controls [33]. In this study we have investigated the functional capacity and cytokine release profile of peripheral blood mononuclear cells (PBMCs) in response to CMV and Aβ antigen challenge as well as the correlation to *ApoE* genotype and systemic inflammatory biomarkers.

## Materials and Methods

### Subjects

From a previously described cohort of patients with AD and ND controls [33], all HLA-A02 positive subjects were included in this study rendering a total of 30 AD patients and 35 ND controls. All study participants’ dementia status was blinded during the laboratory work.

Informed consent was obtained in writing from all study participants, together with written consent from a close relative if there was any uncertainty on whether the subject was capable of providing informed consent him- or herself. The study was approved by the Regional Ethical Review Board in Uppsala, Sweden.

### Preparation of Aβ Protofibrils

Synthetic Aβ1-42 wt (American Peptide Company Inc., Sunnyvale, CA, USA) was dissolved in 10 mM NaOH, diluted in 10x PBS to 443 mM (2 mg/ml) and incubated for 30 min at 37°C. The preparation was centrifuged for 5 min at 17 900×*g* to remove any insoluble fibrillar aggregates and analysed with an Aβ protofibril specific ELISA [34] to confirm that Aβ protofibrils were formed.

### Sampling and Sample Preparation

PBMCs were isolated from blood samples, acquired at the Memory Clinic at the Department of Geriatrics in Uppsala University Hospital, and frozen in accordance with a previously described protocol [33]. *ApoE* genotyping and measurement of C-reactive protein (CRP) was performed at the Department of Clinical Chemistry and Pharmacology at Uppsala University Hospital.

A medium consisting of RPMI-1640 with 10% heat inactivated FBS, 1% penicillin-streptomycin, 1% HEPES, 0.5% L-glutamine, and 0.04% β-mercaptoethanol, was used during all steps. Each PBMC batch of 5×10^6^ cells per subject was thawed in a 37°C water bath and the cell suspension was thereafter immediately washed in 10 ml supplemented medium and centrifuged at 400×*g* for 5 minutes. Next, the cell medium was decanted and cells resuspended and transferred to a flat-bottom 24-well plate. Supplemented medium was added to a total volume of 2 ml per sample well. The plate was incubated in a 37°C, 5% CO_2_, humidified atmosphere for 3 h.

After resuspension, cells were visually counted and assessed for viability. Each sample was divided into eight aliquots containing 10^5^ viable cells, which were transferred to a 96-well round-bottom plate and stimulated in duplicates with either PepMix HCMVA pp65 (JPT Peptide Technologies, Germany), Human T-Expander anti-CD3/CD28 Dynabeads (Invitrogen, California, USA), Aβ protofibrils or left without antigen stimulation as a negative control. CMV pp65 was titrated in accordance with the manufacturer’s recommendations, anti-CD3/CD28 beads were used in a 4∶1 bead:cell ratio and Aβ protofibrils were used in a final concentration of 0.1 µM. All samples were supplemented with corresponding amounts of DMSO to compensate for the DMSO needed to dissolve the pp65 peptide mix.

Samples were incubated for 16 h in a 37°C, 5% CO2, humidified atmosphere, followed by centrifugation for 5 min at 250×*g* after which the supernatants were stored in −70°C until cytokine analysis. Assay incubation time was based on the QuantiFERON CMV protocol, optimised for the kinetics of IFN-γ response [35].

### Cytokine Quantification Assay

The cytokine analysis was performed using a MAGPIX instrument (Luminex Corporation, Texas, USA), xPONENT software (version 4.2.1324.0) and a MILLIPLEX MAP Human Cytokine/Chemokine Panel (EMD Millipore, Massachusetts, USA) detecting GM-CSF, IFN-γ, IL-1β, IL-2, IL-4, IL-5, IL-6, IL-8, IP-10 and TNF-α. The cytokine panel was designed to provide a measure of both Th1 and Th2 inflammatory response. All samples, standards and controls were run in duplicates and handled in accordance with the manufacturer’s protocol. Washing steps were performed twice. The system was set to analyse 100 µl of sample per well and collect a minimum of 100 beads per region.

### Statistics

R version 3.0.1 (The R Foundation for Statistical Computing) was used for statistical analysis. All comparisons were made using the non-parametric Mann-Whitney U-test. All p-values below 0.05 are presented in their uncorrected form, together with a Bonferroni correction for the internal multiplicity of 10 corresponding to the number of cytokines in the assay.

Box plots were defined with boxes containing quartiles 2 and 3 and whiskers displaying quartiles 1 and 4, excluding any outliers outside 1.5 times the interquartile range. For all logarithmic plots of cytokine concentrations, values below 0.1 pg/ml were set to 0.1 to allow logarithmic visualisation.

## Results

### Background Data

The epidemiological and laboratory background data of the AD and ND subjects are described in Table 1. As a small number of analytic results were returned blank or erroneous, the mean values have been calculated on the available data.

**Table 1:** Summary of baseline characteristics in patients with Alzheimer’s disease (AD) and non-demented controls (ND).

Continuous data reported as mean (Standard Deviation)	AD (N = 30)	ND (N = 35)
Age, years	78.3 (6.54)	74.1 (6.81)
Gender, male/female	17/13	16/19
Mini-mental State Examination score	19.8 (5.19)	NA
*APOE* ε4 allele carriers, hetero-/homozygote	17/2	13/1
CMV IgG positive	87% (n = 26)	86% (n = 30)

NA = Not Available.

### Cytokine Quantification

**Figure 1:**
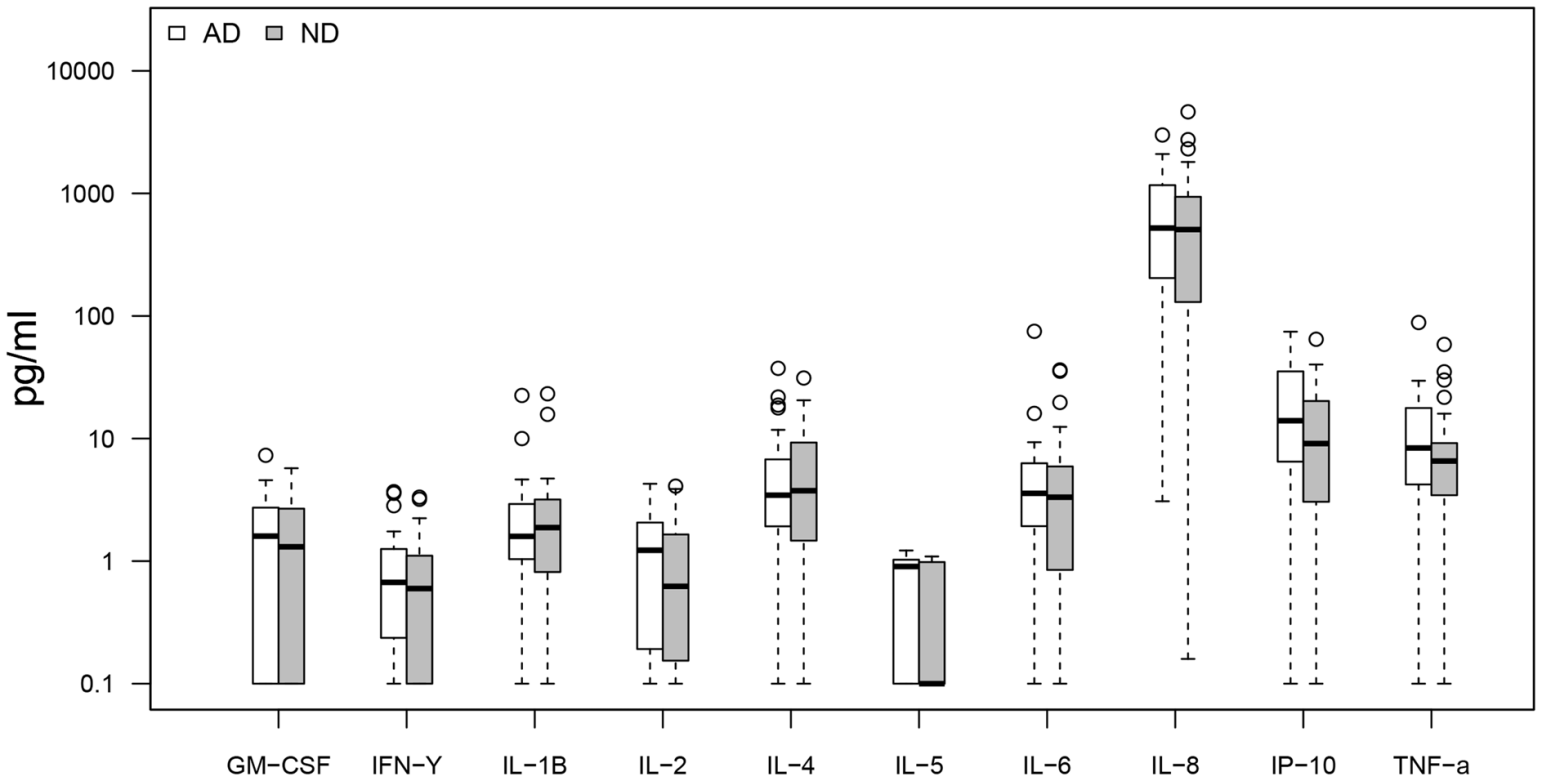
AD vs. ND, negative control. No significant differences in baseline cytokine secretion between AD and non demented (ND) subjects. Only CMV seropositive subjects were included.

**Figure 2:**
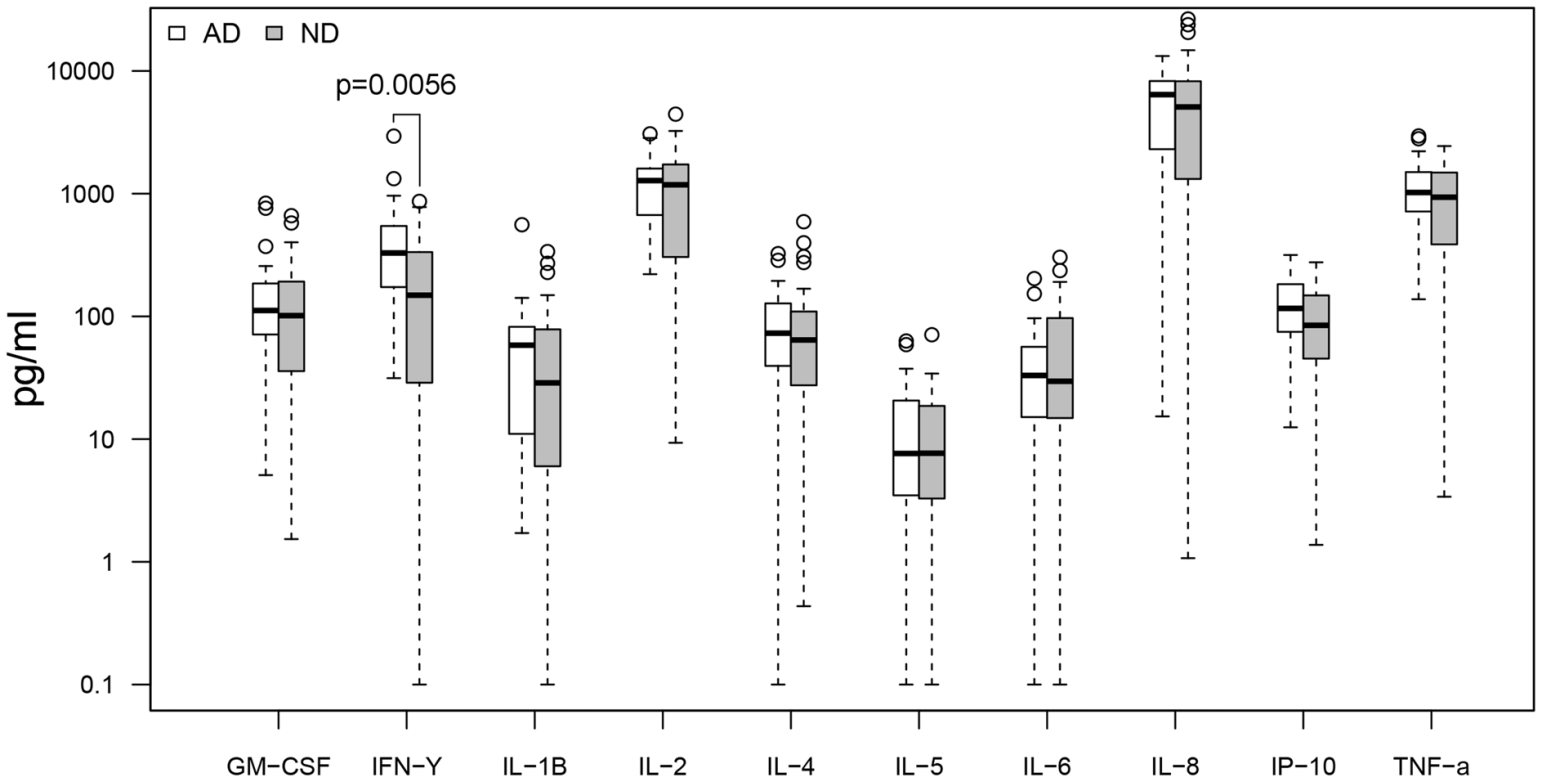
AD vs. ND, anti-CD3/CD28 stimulation. AD patients showed higher levels (p = 0.0056 or 0.056 with Bonferroni correction) of IFN-γ upon CD3/CD28 stimulation compared to non demented (ND) controls. Only CMV seropositive subjects were included.

**Figure 3:**
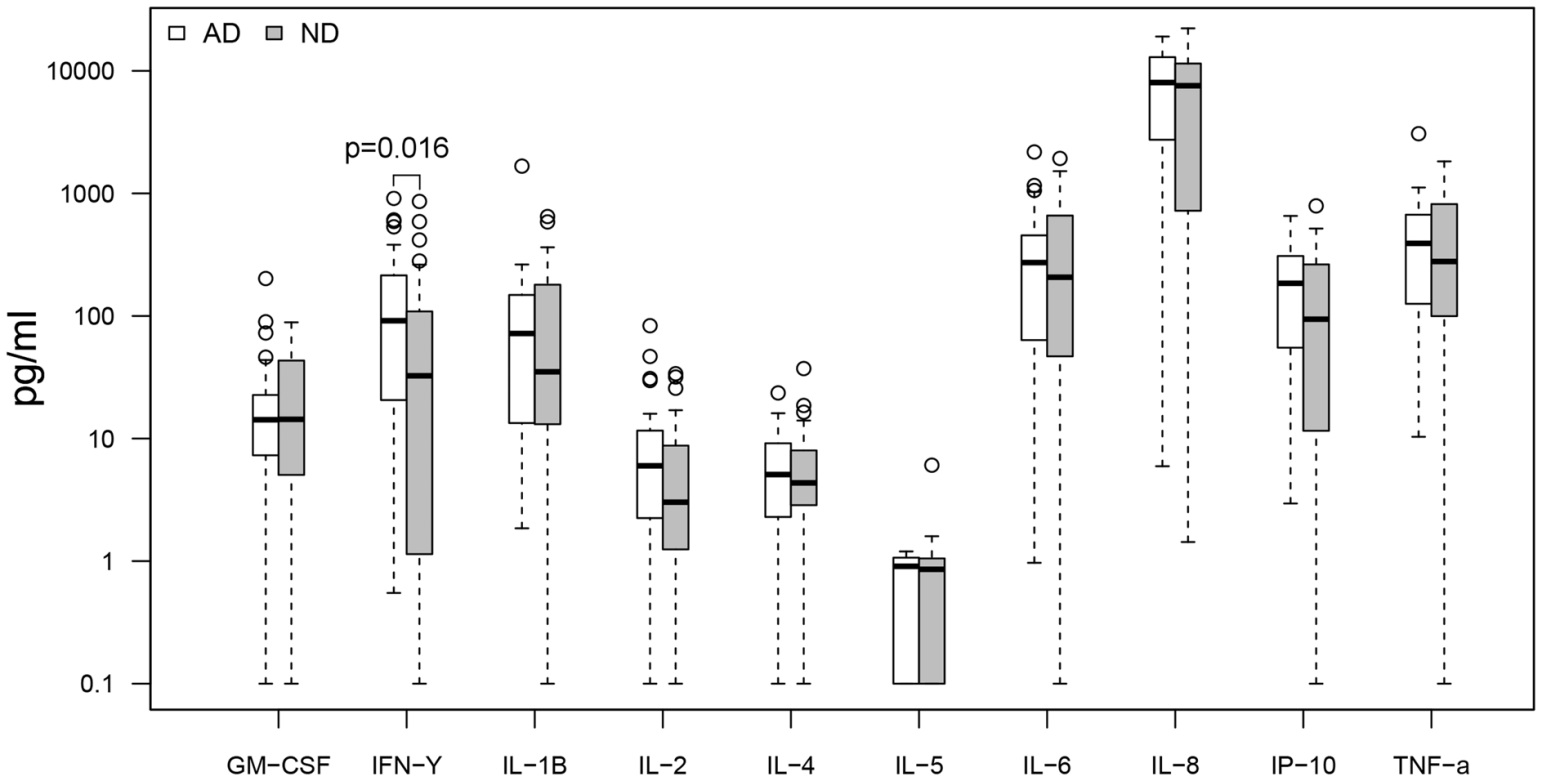
AD vs. ND, CMV pp65 stimulation. Higher levels (p = 0.016 or 0.16 with Bonferroni correction) of IFN-γ upon pp65 stimulation in AD patients compared to non demented (ND) controls. Only CMV seropositive subjects were included.

**Figure 4:**
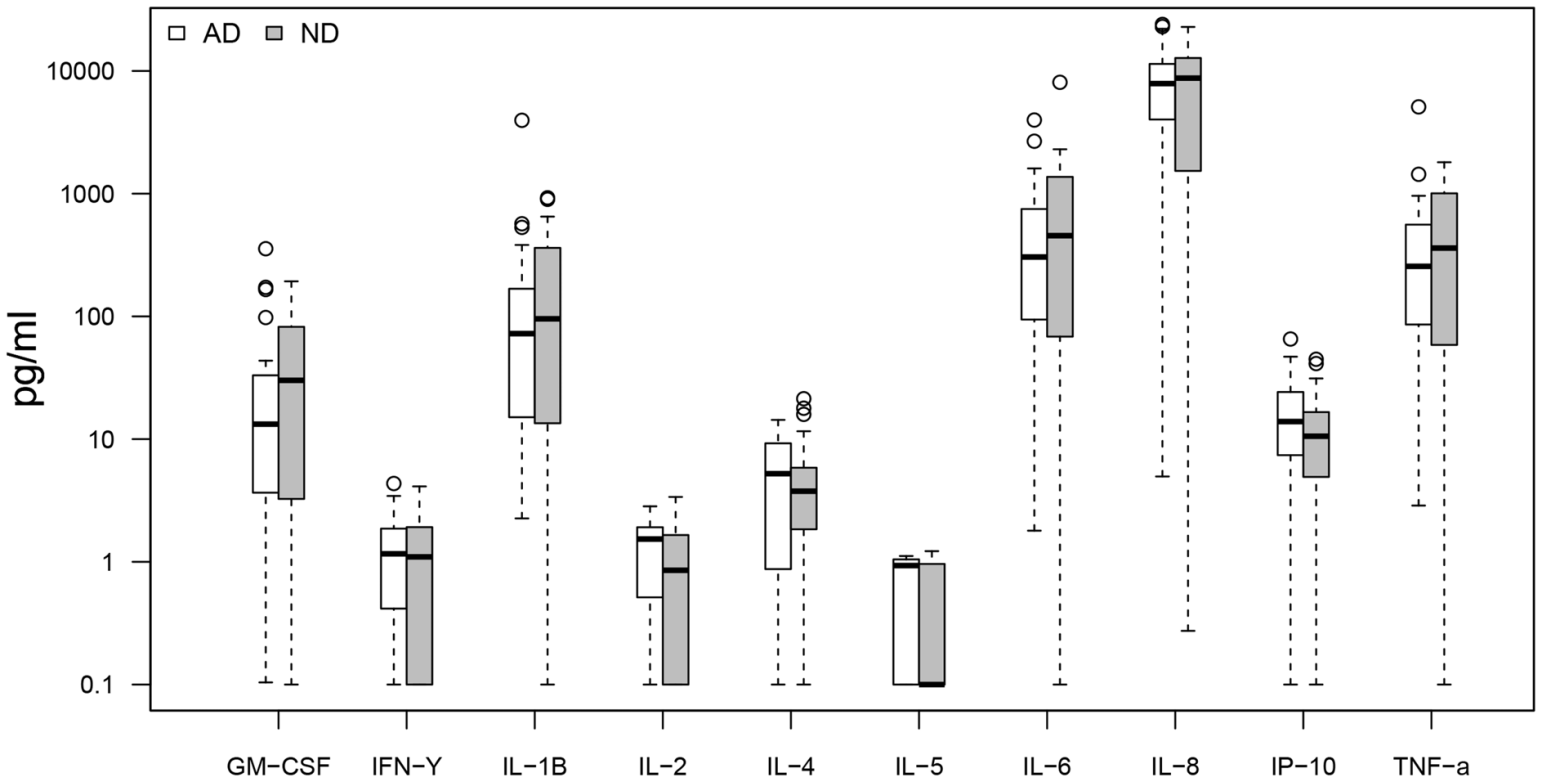
AD vs. ND, amyloid beta protofibril stimulation. No significant differences in cytokine secretion between AD patients and non demented (ND) controls upon Aβ protofibril stimulation. Only CMV seropositive subjects were included.

Cytokine response profiles were compared between CMV seropositive AD (n = 26) and ND subjects (n = 30) (Figures 1–4). AD subjects presented with similar baseline cytokine levels as ND controls, but showed a higher IFN-γ response upon stimulation with both anti-CD3/CD28 Dynabeads and CMV pp65. There was no difference in cytokine response between AD and ND subjects upon Aβ protofibril stimulation.

**Figure 5:**
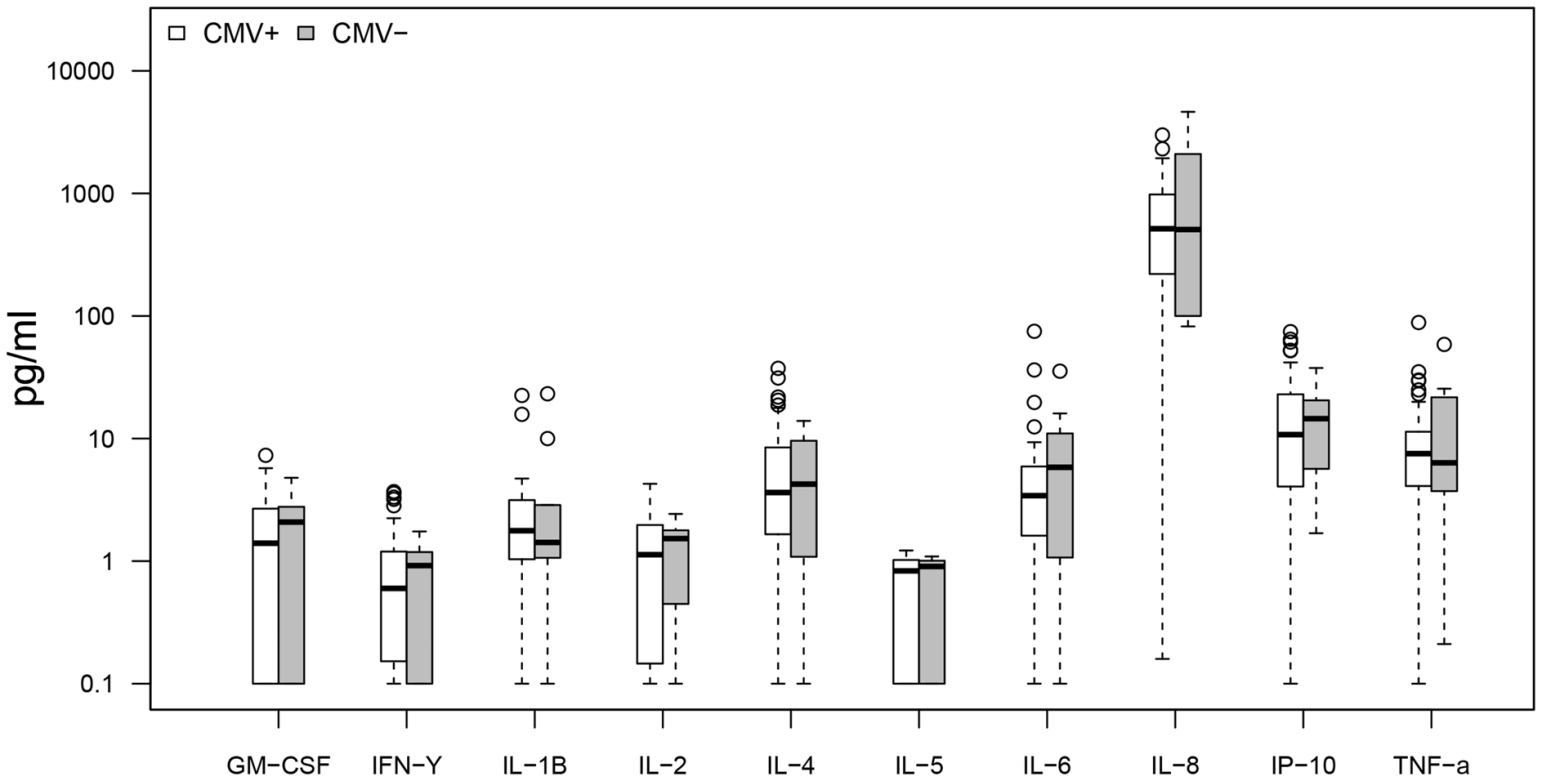
CMV+ vs. CMV−, negative control. No significant differences in baseline cytokine secretion between CMV seropositive and seronegative subjects. All subjects included regardless of dementia status.

**Figure 6:**
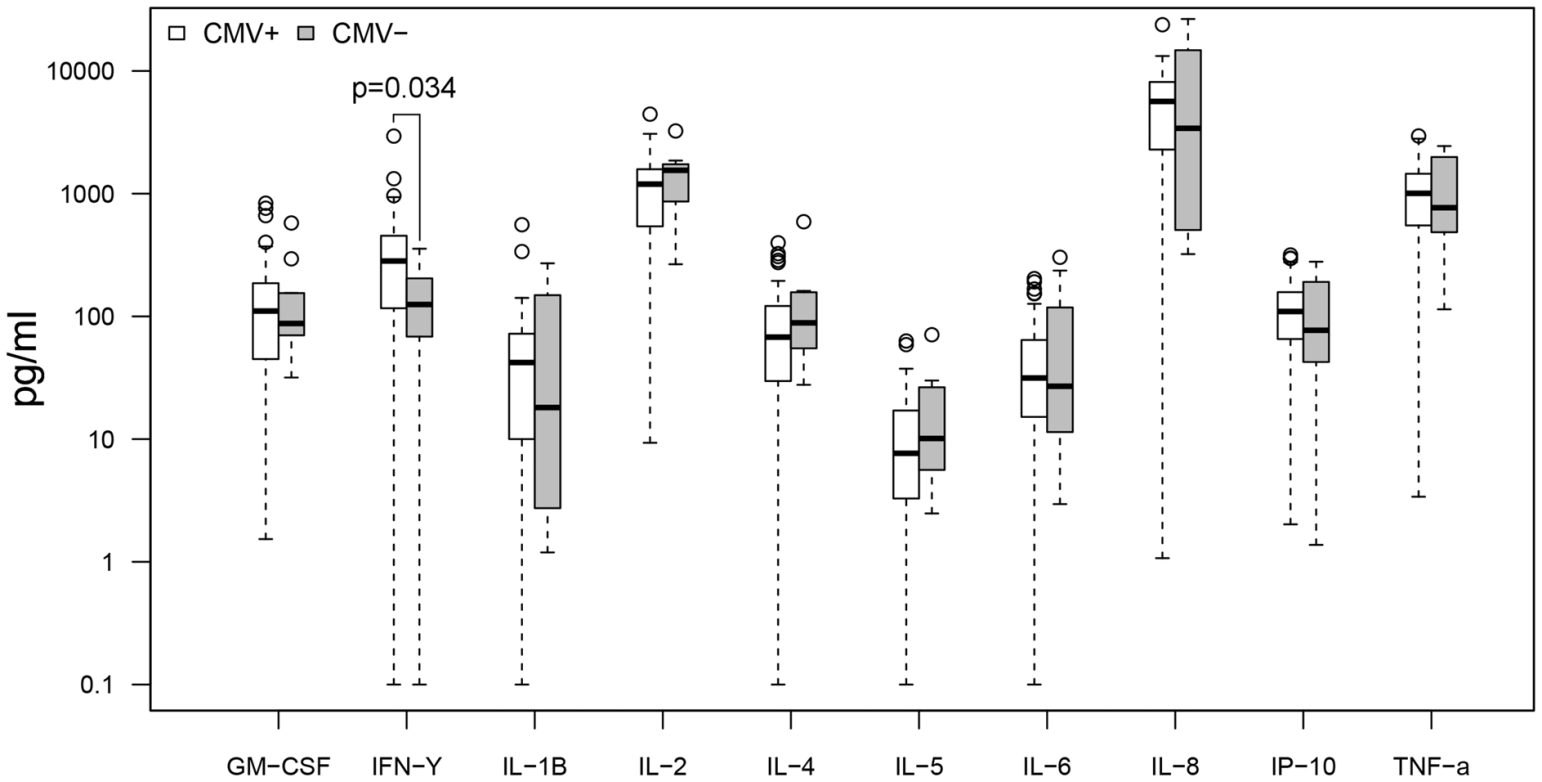
CMV+ vs. CMV−, anti-CD3/CD28 stimulation. Higher levels (p = 0.034 or 0.34 with Bonferroni correction) of IFN-γ in CMV seropositive subjects compared to seronegative subjects, upon CD3/CD28 stimulation. All subjects included regardless of dementia status.

**Figure 7:**
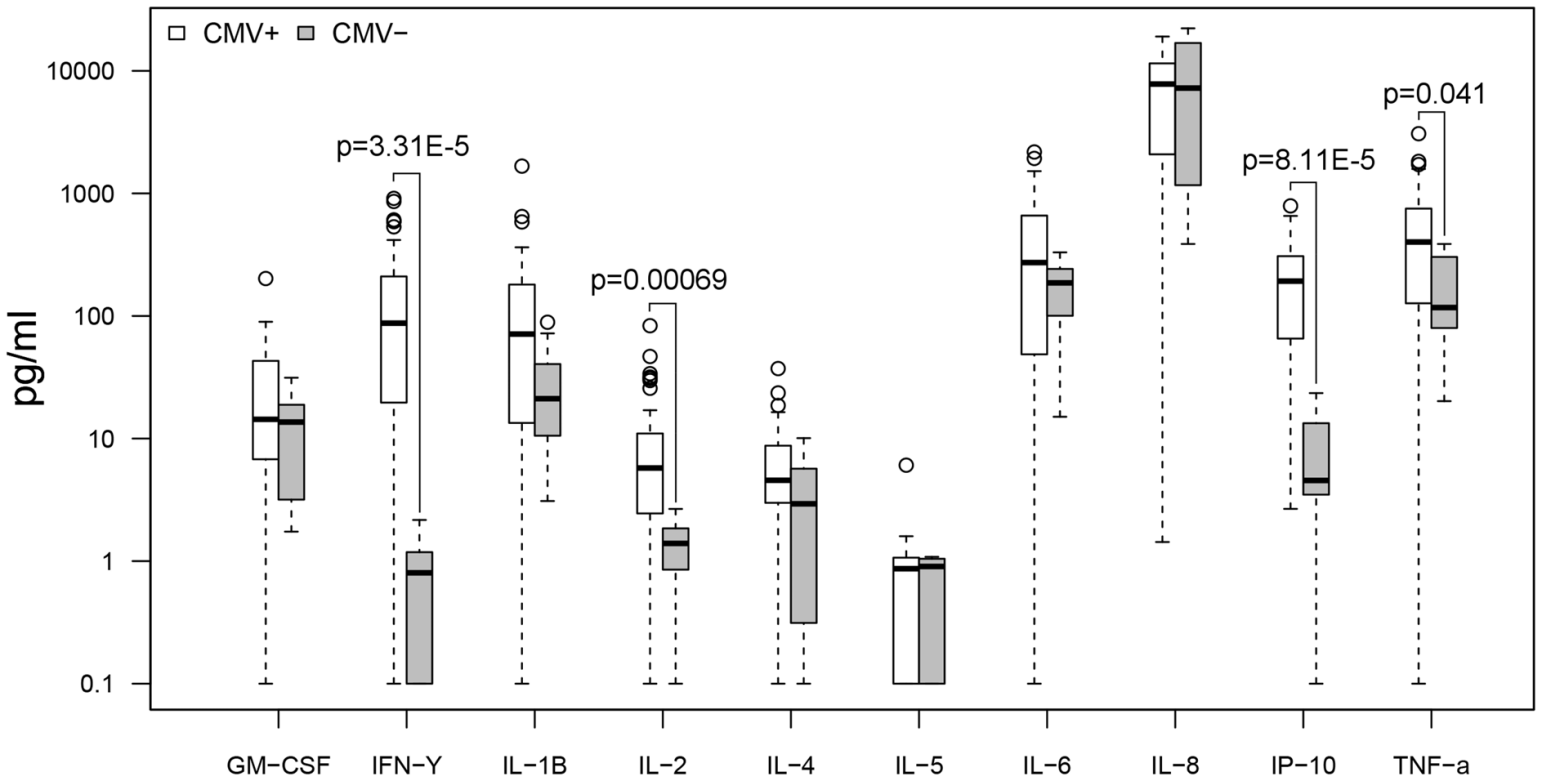
CMV+ vs. CMV−, pp65 stimulation. Higher levels of IFN-γ (p = 3.31E-5 or 0.00033 with Bonferroni correction), IL-2 (p = 0.00069 or 0.0069), IP-10 (p = 8.11E-5 or 0.00081) and TNF-α (p = 0.041 or 0.41) in CMV seropositive subjects compared to seronegative subjects. All subjects included regardless of dementia status.

**Figure 8:**
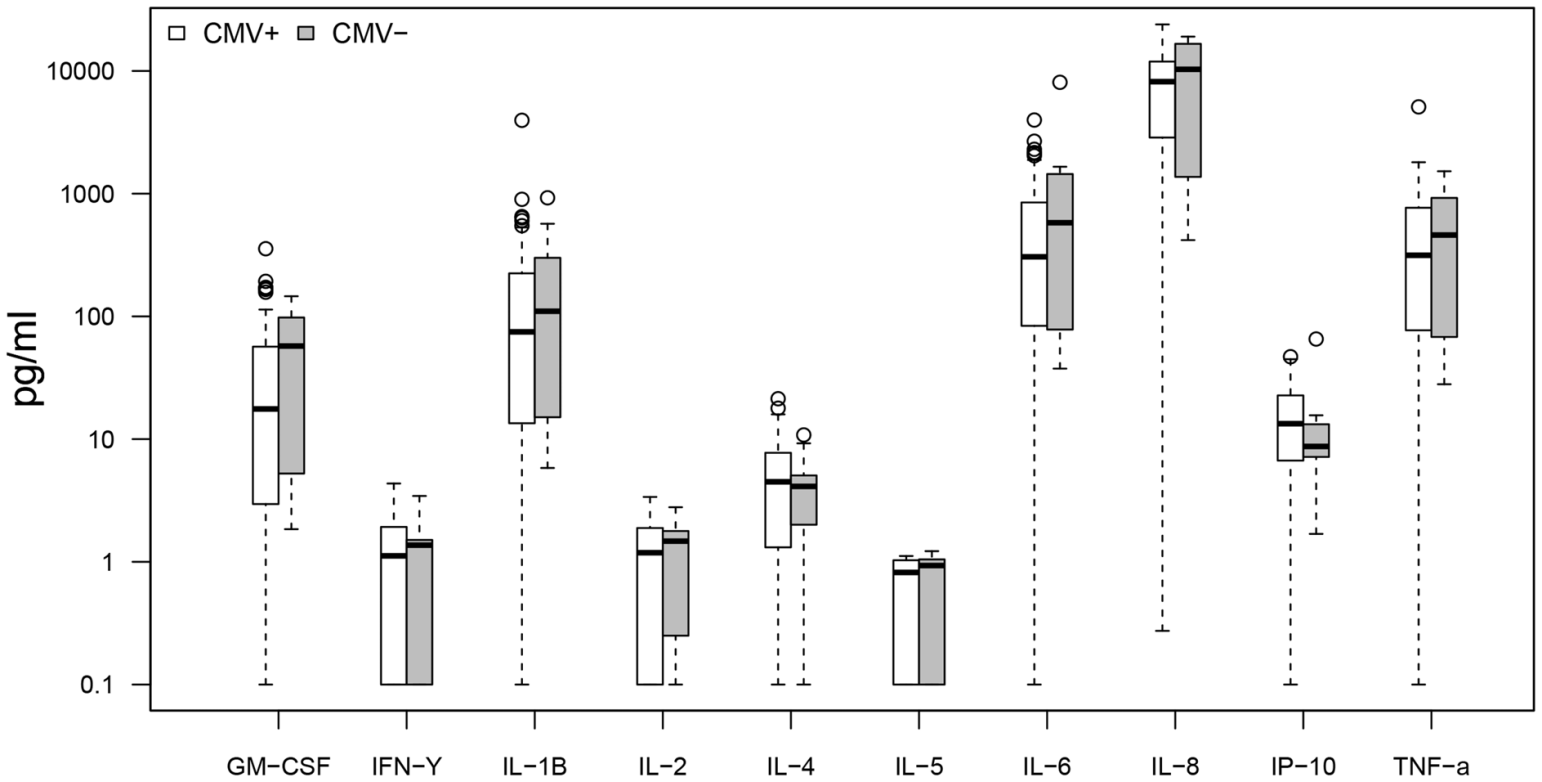
CMV+ vs. CMV−, amyloid beta protofibril stimulation. No significant differences in cytokine secretion between CMV seropositive and seronegative subjects upon Aβ protofibril stimulation. All subjects included regardless of dementia status.

When comparing CMV seropositive (n = 56) and seronegative (n = 9) subjects regardless of dementia status (Figures 5–8), there were no differences in baseline cytokine levels or response upon Aβ protofibril stimulation. However, CMV seropositive subjects presented with a higher IFN-γ response upon stimulation with anti-CD3/CD28 Dynabeads. Moreover, stimulation with CMV pp65 resulted in a strong response with a 200-fold increase in levels of IFN-γ, 8-fold increase in IL-2, 30-fold increase in IP-10 and 3-fold increase in TNF-α in the CMV seropositive group compared to the seronegative group.

**Figure 9:**
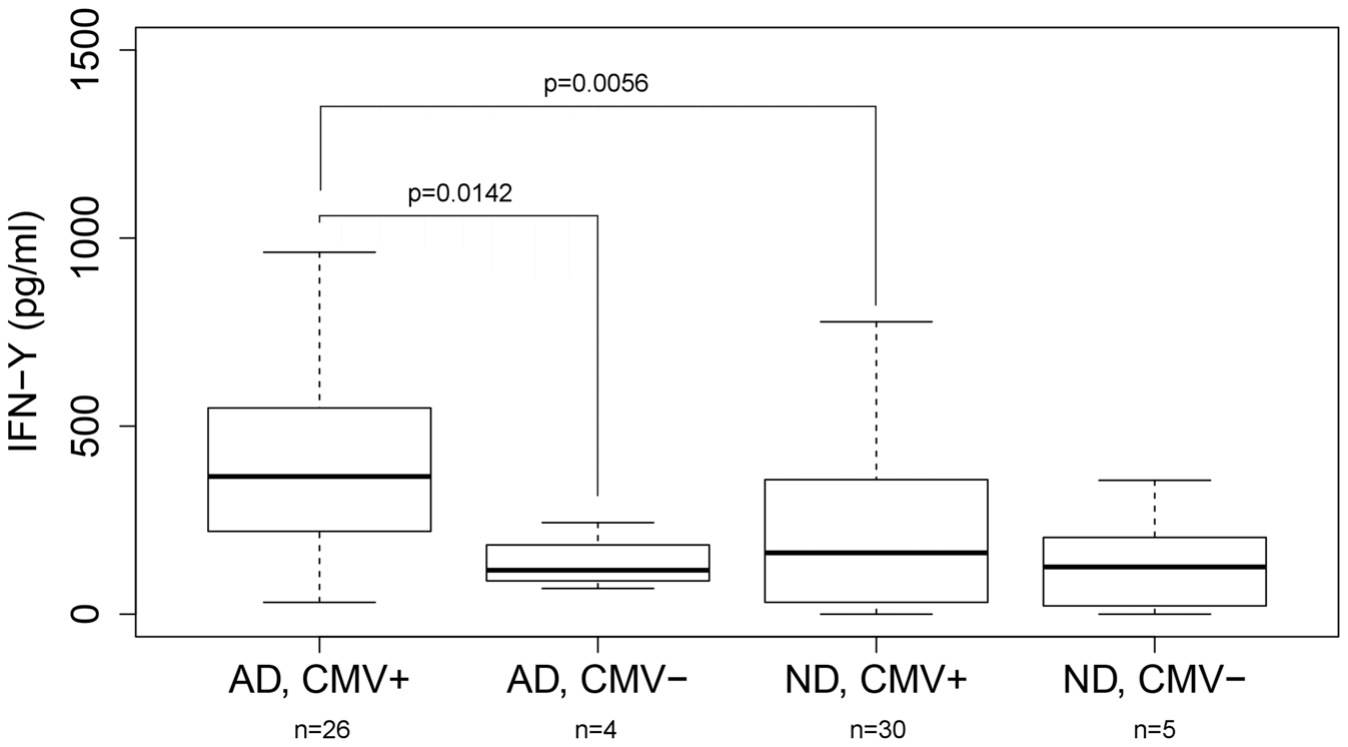
Sub-group analysis of IFN-γ response upon anti-CD3/CD28 stimulation stratified by dementia and CMV status. Significantly higher response in the AD CMV seropositive group, compared to both AD CMV seronegative subjects (p = 0.0142 or 0.14 with Bonferroni correction) and non-demented CMV seropositive subjects (p = 0.0056 or 0.056). Outliers excluded from plot, but included in the statistical analysis.

To further investigate how IFN-γ release upon anti-CD3/CD28 stimulation correlated to dementia status and CMV infection, the interaction was analysed by separating the AD and ND groups by their CMV serostatus (Figure 9). AD CMV seropositive subjects presented with higher IFN-γ response, both compared to AD CMV seronegative and ND CMV seropositive subjects, indicating a potentiation of the inflammatory response in CMV infected AD patients.

### Correlation between PBMC Reactivity, Systemic Inflammation and ApoE Genotype

**Figure 10:**
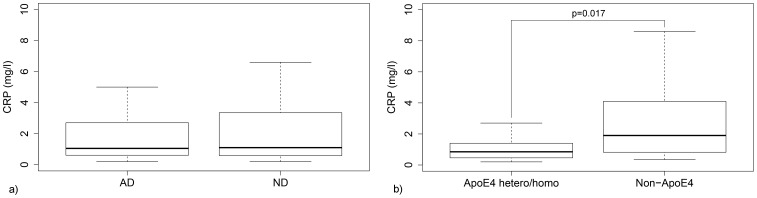
Dementia status and Apolipoprotein E genotype vs. plasma CRP. **a)** No difference in plasma CRP levels between AD and ND groups. **b)** Hetero- and homozygote *ApoE ε4* allele carriers present with lower plasma CRP compared to non-carriers (p = 0.017). Outliers excluded from plots, but included in statistical analysis. Both CMV seropositive and seronegative subjects included.

To investigate whether the PMBC reactivity was related to general systemic inflammatory response, the IFN-γ release data were plotted against plasma CRP revealing no significant correlation (data not shown). Plasma CRP was also compared between AD and ND subjects, rendering no difference between the groups. When comparing *ApoE ε4* allele carriers and non-carriers regardless of dementia status, subjects with one or two ε4 alleles presented with significantly lower CRP levels (Figure 10).

## Discussion

In this study, CMV seropositive AD and ND groups showed similar cytokine levels at baseline, indicating no increase in PBMC baseline inflammatory response in AD subjects. However, AD subjects showed a stronger IFN-γ response to both anti-CD3/CD28 and CMV pp65 compared to ND subjects, which we believe is the result of a more inflammation-prone T-cell phenotype that could also affect levels of inflammation in specific organs without producing significantly higher levels of inflammatory biomarkers in peripheral blood. When analysing the IFN-γ response in subgroups of CMV seropositive and negative AD and ND patients, our data suggest that the increased Th1/Tc1-like proinflammatory response in the AD group is only due to the strong reactivity in the CMV seropositive AD individuals, something not seen in CMV seropositive ND or CMV seronegative AD subjects. Stimulation with Aβ protofibrils induced a comparable response between AD and ND groups, independent of CMV serostatus and with a response pattern showing no increase in IFN-γ but strongly elevated levels of GM-CSF, IL-1β, IL-6, IL-8 and TNF-α suggesting that much of this reactivity could originate from antigen presenting cells such as monocytes.

The PBMC inflammatory response has been previously investigated in an AD study, where demented subjects presented with increased baseline cytokine secretion compared to ND elderly. Furthermore, AD subjects presented with higher IL-1β compared to ND elderly upon stimulation with anti-CD3/CD28 and very little Aβ stimulatory response was seen in either group [32]. The results in our study are somewhat in conflict to these previous findings, but possible explanations include differences in methodology affecting cell viability as well as differences in group epidemiology.

When strictly applying the Bonferroni correction needed to adjust for the multiplicity of 10 parallel cytokine measurements, the difference in IFN-γ levels did not quite reach statistical significance. However, as the difference in IFN-γ was reproduced between our different assay configurations we believe that the finding is not coincidental and that the uncorrected p-values of 0.0056 and 0.016 are relevant when comparing AD and ND groups after anti-CD3/CD28 and CMV pp65 stimulation. However, additional studies are needed to confirm these results and should preferably be designed to optimize statistical power by targeting a more limited set of cytokines and having equal group sizes in terms of CMV serostatus.

Our data showed a strong CMV-specific pp65-induced response when comparing CMV positive versus negative subjects, in accordance with results from the commercially available QuantiFERON CMV kit [35], that validates the biological relevance of our assay. Moreover, there was no correlation between PBMC IFN-γ secretion and plasma CRP levels, indicating that a more inflammation prone cellular response does not directly translate into an increased baseline systemic inflammation. As expected, the *ε4* allele was found to be more common in the AD group. Our data also confirms previous studies showing that the *ApoE ε4* allele correlates with lower levels of CRP, which could be due to down-regulation of the mevalonate pathway [36], [37]. However, it is still not clear if this affects the risk of AD. Systemic inflammation is generally considered a risk factor for dementia, but in studies of *ApoE ε4* allele carriers CRP has been inversely correlated with both all-cause dementia and levels of CMV IgG [38], [39].

In this study, all patients had clinically manifest AD and it could very well be that the most interesting phase of the pathophysiological process had passed, leaving only inflammatory traces that are less specific than what we would find in subjects at an earlier stage of disease. Furthermore, although antigen stimulation of unsorted PBMCs and subsequent supernatant analysis has the advantage of allowing quantification of the total cytokine response regardless of cell type, similar to *in vivo*, it also leads to difficulties in interpreting reactivity from separate cell types as the cytokine profiles are cumulative.

We believe that these results provide further insight into how the immune response to CMV infection interacts with the aging immune system in AD, but it is still not clear if and how CMV infection connects to the pathogenesis. As there is more direct evidence of HSV-1 residing in brain tissue of AD patients, and this virus recently was epidemiologically linked to negative cognitive effects in subjects of all ages [40], it could be that CMV-induced dysregulation of the inflammatory response increases the risk of AD by allowing more frequent or prolonged episodes of HSV-1 reactivation. That would also be consistent with results from Stowe et al, showing that HSV reactivation is more common in CMV seropositive subjects [41]. Another hypothesis is that CMV infection affects AD pathogenesis more directly by increasing local inflammatory response against a wide variety of antigens, possibly including Aβ. This could both induce the production of the amyloid precursor protein as well as the formation of neurotoxic protofibrils, thereby promoting cognitive decline [42]. To better estimate the clinical implications of our findings, further studies with a prospective design are needed, analysing CMV serostatus, systemic inflammation and cellular immune response, in subjects at prodromal disease stages.
